# Using deep learning to predict ideology from facial photographs: expressions, beauty, and extra-facial information

**DOI:** 10.1038/s41598-023-31796-1

**Published:** 2023-03-31

**Authors:** Stig Hebbelstrup Rye Rasmussen, Steven G. Ludeke, Robert Klemmensen

**Affiliations:** 1 Department of Political Science, Århus University, Aarhus, Denmark; 2 Department of Psychology, University of Southern Denmark, Odense, Denmark; 3 Department of Political Science, Lund University, Lund, Sweden

**Keywords:** Human behaviour, Psychology

## Abstract

Deep learning techniques can use public data such as facial photographs to predict sensitive personal information, but little is known about what information contributes to the predictive success of these techniques. This lack of knowledge limits both the public’s ability to protect against revealing unintended information as well as the scientific utility of deep learning results. We combine convolutional neural networks, heat maps, facial expression coding, and classification of identifiable features such as masculinity and attractiveness in our study of political ideology in 3323 Danes. Predictive accuracy from the neural network was 61% in each gender. Model-predicted ideology correlated with aspects of both facial expressions (happiness vs neutrality) and morphology (specifically, attractiveness in females). Heat maps highlighted the informativeness of areas both on and off the face, pointing to methodological refinements and the need for future research to better understand the significance of certain facial areas.

## Introduction

What can we learn from a face, and what is it about the face that provides us information? Everyday judgments made by untrained laypeople have some degree of accuracy in predicting a range of psychological characteristics, including personality, intelligence, political ideology, and sexual orientation^1–4^. In these and other areas, however, deep learning approaches have provided significant predictive improvements over layperson judgments^5–7^.

The success of deep-learning approaches for predicting psychological characteristics is not only socially important given privacy protection concerns, but scientifically intriguing as well. However, to provide deeper psychological insights and to help the public identify ways to protect sensitive information, the use of deep learning approaches should not stop with successful classification. We should also explore *what* it is about a face that communicates the sensitive information. Are deep learning approaches simply confirming and independently detecting previously-posited facial indicators of a given characteristic, or are they picking up on salient facial differences which were previously unrecognized?

The present study explores the relationship between faces and ideology using a large, publicly-available sample containing both facial photographs and ideological data—namely, 3233 Danish political candidates for local office. We use a wide range of techniques, including convolutional neural networks, heat maps, analyses of facial expressions, and assessments of physical characteristics such as masculinity and beauty. By integrating these approaches we go beyond identifying the degree to which faces connect to ideology in two ways: First we identify specific features of the face that connect to the model’s predictions for political ideology. Second, we explore limitations to existing approaches which lead to inflated estimates of the association between faces and psychological characteristics.

### Faces and ideology

The study of faces in politics has a long history. Early work found that voters perceive their preferred candidates to be more attractive than opposing candidates^8^. Voter perceptions are themselves politically consequential: For example, faces evaluated as more competent were substantially more successful in an election^9^, and attractiveness provides electoral bonuses as well^10^.

This literature has generally posited the relevance of faces to politics to be limited to *subjective impressions* of those faces^11^. A recent study^7^ strongly refocused the argument by demonstrating that algorithmic classification of faces led to substantial accuracy when predicting ideology. This study’s focus, however, was to demonstrate the significant privacy threat posed by the intersection of deep learning techniques and readily-available photographs^7^. This seminal work highlighted the need for further study of the topic, particularly with respect to identifying what features of a photograph contribute to successful algorithmic classification of faces.

Such investigations are in part needed simply to confirm the relevance of *faces* per se. No study has yet employed heat-mapping to identify which segments of facial photographs are informing the classifications provided by the deep learning algorithms when it comes to faces being classified as being rightist or leftist. Because standard cropping procedures for facial photographs (including those employed in prior deep learning facial studies) leave non-facial information in the image, heat-mapping is important to discern whether information beyond the face is informing the classification of faces.

The use of heat-mapping extends beyond demonstrating potential needs for methodological refinements, however, in also highlighting which facial features are the most informative. This facilitates a second area of that needs exploration: W*hy* are faces informative for ideology? That is, what information is a neural network detecting when it learns how to correctly identify which faces belong to those endorsing a given ideology? The social importance of the question is clear from a privacy protection perspective: Facial photographs are commonly available to potential employers, and those involved in hiring decisions self-declare a willingness to discriminate based on ideology^12,13^. Members of the public may thus be aided by recognizing what elements of their photographs could affect their chances of employment. But understanding the facial elements responsible for successful predictions of associations between faces and ideology is also important for the study of ideology. Knowing *that* faces detectably differ based on ideology is undeniably important but identifying the specific features of interest may help to illuminate the origins of ideological differences.

Several plausible candidates exist. Some of these concern facial morphology, i.e., stable physical characteristics of the face. For example, politicians on the right have been found to be more attractive than those on the left^10^. Higher right-wing attractiveness scores were also observed among young (male) adults in the general population^10^, though not among pundits^14^. Masculine facial features may also be relevant: Masculine characteristics such as upper body strength appear to be associated with conservative economic preferences^15^, and a preference among conservative voters for masculine-looking males is well established^16,17^.

Facial expressions may also be relevant. A recent study^7^ reported that computer-coding of facial expressions were associated with self-reported ideology, with those on the left showing more surprise and less disgust. This study^7^ focused on quantifying the degree of threat to privacy, reported only the *degree* to which other facial expressions could independently facilitate the classification of ideology, and not the *direction* of any other associations.

However, the observation of an *actual* association between any given expression or aspect of facial morphology does not entail that this association is being detected by the algorithmic processes used to classify the faces by ideology. No study has yet tested whether model-implied ideology correlates with *any* facial characteristic, whether morphological or expressive. As such, the degree to which these features are informing deep-learning approaches and thus represent a privacy threat needs further consideration.

### The present study

We begin by exploring how computational neural networks classify political ideology from a single facial photograph. We then use heat maps to examine how non-facial information contributes to these classifications and to identify facial features of interest for future study. We next examine how a range of characteristics of faces—masculinity, attractiveness, and expressions—connect to model-predicted ideology. Our goal is thus not maximizing accuracy per se—had we explored every model architecture and every set of hyperparameters, it is likely that the accuracy presented here would be increased. But the question of *whether* facial photographs can predict whether someone is leftist or rightist is well answered in the affirmative^7^. We thus instead explore *why* those predictions are possible. The present work is therefore contributing to “interpretable AI”. We add to “interpretable AI” by using theory-guided facial features to seek to identify why deep learning can successfully classify faces by ideology. Thereby we move beyond the many such studies which seek to peek within the “black box” of deep learning by using features of the model itself^18^. The present work is thus part of the social data science tradition, in which machine learning methods are combined with theories and empirical results from traditional social science.

## Methods

### Data and code availability statement

The data has been processed in accordance with Danish and European GDPR regulations according to the University of Aarhus Data Controller Unit. The data is based on publicly available data at the website from the Danish Broadcasting Corporation (DR). Those who wish to access the data should contact DR at https://dr.custhelp.com/app. Scripts to redo the analyses will be made available on OSF upon publication. The study was not preregistered. The informed consent of participation and publication of the facial images presented in this article was obtained from the participants.

### Sample

#### Municipal candidates

Our primary dataset consists of 5230 facial photographs of political candidates from the 2017 Danish Municipal election. These photos are a publicly-available resource, provided for use in the public sphere by the candidates themselves to the Danish Broadcasting Cooperation (DR). Danish municipal elections take place in a non-polarized setting. Candidates running for office in these elections have not been highly selected through competitive elections in party organizations or by participation in high-stakes elections, and they are thus described as the “last amateurs in politics” by Danish political scientists^19^.

Each candidate’s ideology (dichotomously scored as left- or right-wing) was assigned based on the party label under which the candidates ran—see the list of specific parties in Supplementary Materials 1. Members of local parties with less-defined ideologies could not be readily assigned an ideological score and so were excluded prior to any analyses, leaving 4647 candidates, of which 1442 were female. These images were then manually inspected by an author blind to the candidate’s name or party. Additional exclusion criteria applied at this time included: (1) the candidate’s face was either not located during pre-processing, not shown in the photo, or not provided in sufficient resolution; (2) the photo was not in color (which would prevent pre-processing all images via gray-scaling in the same way); (3) the candidate was the candidates who did not appear to our rater to be of European ethnic origin, as this small subset (118 individuals) skewed very highly towards left-wing parties, with 2.5 × more representation there than among right-wing parties.

After applying these exclusion criteria we had 3288 candidate photos remaining—this is our full primary sample. We then separated the males and females before using R to sample each gender into training, validation and test samples with probabilities of 0.7, 0.15 and 0.15. *N*s for each sample are provided in Table S1.

Because beards can impair some of the subsequent analyses (such as the detection of facial expressions^20,21^, we produced a “reduced” version of the male training, validation, and test samples in which those with beards (*N* = 657) were dropped. This sample is used for our analyses that involve “identifying salient characteristics” (see below).

#### Replication sample: Danish parliamentarians

To provide a second test for the accuracy of our algorithmic predictions of ideology based on faces, we obtained an additional sample, namely Danish Parliamentarians. The sample was created following the procedure described for the primary sample, omitting the final two steps. That is, other than separating males and females no division of the sample was undertaken. Instead, we used the entire sample as a test sample. Because we used this small participant pool to evaluate our model’s predictive accuracy and not to evaluate facial morphology or expressions, we retained those with beards.

### Image preparation

To ensure that only the faces and not the use of a certain background or a certain set of clothes are biasing our results, we used the dlib library implemented in Python to locate and crop the face. In addition we used the Python OpenFace library^22^ to align the images such that faces are centered and have the same rotation.

**Figure 1:**
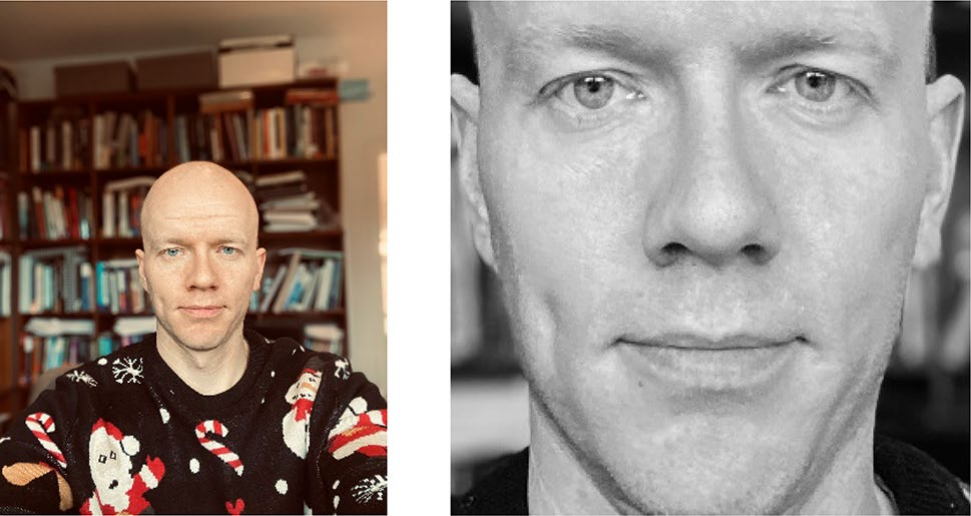
Example of cropping an image.

Danish political alignment includes a color element (with left- and right-wing parties denoted by red and blue colors, respectively). To prevent color choices from influencing model results we used OpenCV to turn all photos into black and white images. An example image that has been cropped and manipulated as described is provided in Fig. 1. This cropping is all that was performed prior to our first analyses, though as described in the results section, we then found an additional cropping step (described further there) was required.

### Measures

#### Facial expressions

We used the Face API from Microsoft Azure's Cognitive Services to identify facial expressions. Facial photographs are assigned a score for how much the face in question resembles each of a list of emotional states—e.g., happiness, sadness, surprise. Descriptive statistics for our full primary sample (Table 1) indicated that faces were overwhelmingly scored as indicating happiness (80%), with neutrality indicated as the second most frequent (19%). The remaining expressions were indicated to be infrequent (ranging of 0.00 to 0.01%). While this balance of facial expressions is not necessarily implausible for photos provided in an election context, the results could potentially reflect imperfect scoring procedures of the Face API. An attempt to validate the API found it very successful at identifying happy and neutral facial expressions, though it was less successful for others (anger and surprise), with very unsuccessful identification of still others such as disgust and fear^23^.

**Table 1:** Descriptive statistics for facial expressions.

	Males				Females			
	Mean	SD	Min	Max	Mean	SD	Min	Max
Anger	0.00	0.01	0.00	0.35	0.00	0.00	0.00	0.03
Contempt	0.01	0.03	0.00	0.45	0.00	0.02	0.00	0.25
Disgust	0.00	0.01	0.00	0.10	0.00	0.01	0.00	0.09
Fear	0.00	0.00	0.00	0.02	0.00	0.00	0.00	0.01
Happiness	0.79	0.32	0.00	1.00	0.92	0.19	0.00	1.00
Neutral	0.19	0.30	0.00	1.00	0.08	0.18	0.00	1.00
Sadness	0.00	0.02	0.00	0.35	0.00	0.01	0.00	0.25
Surprise	0.00	0.00	0.00	0.06	0.00	0.00	0.00	0.01

The scores are based on the training samples since this is the largest sample and represents what the model has “learned.” Each face is identified as representing a given expression to a given degree. Thus, a given face could be scored as primarily similar to happiness (0.65), but also similar to a neutral expression (0.35).

#### Facial morphology

##### Masculinity

**Figure 2:**
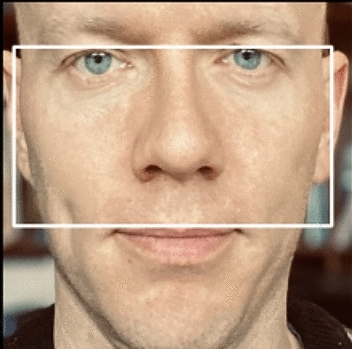
Illustration of fWHR.

We follow established practice and measure masculinity in male candidates, using the facial width-height ratio (fWHR; obtained from https://github.com/TiesdeKok/fWHR_calculator), which is illustrated in Fig. 2. Faces high in fWHR are typically perceived as aggressive and dominant^24^ and as having higher rank and status^25,26^. High fWHR have been found to predict achievements in both the business^27^ and athletic domains^28^.

fWHR scores are obtained by dividing the bizygomatic breadth (the length of the white box) by the distance between the top eyelid and the upper lip (the height of the white box)^28^. Male masculinity scores in the sample averaged 1.96 [*SD* = 0.16].

##### Attractiveness

To predict facial attractiveness scores for our sample we use the publicly-available SCUT-FBP5500 dataset (https://github.com/HCIILAB/SCUT-FBP5500-Database-Release). This dataset consists of 5500 faces rated by 60 raters using a beauty score ranging from 0–4. (Scoring attractiveness as the average value from multiple raters is commonly employed in political science studies on facial attractiveness^10^.

To predict attractiveness scores for our study participants we employ the same CNN model that we used for ideology. The only difference between the network used for predicting ideology and this model is that we are faced with a regression problem instead of a classification problem. Consequently the loss-function is therefore different. We create one model for each gender, using only Caucasians. The model for females had a mean squared error (MSE) of 0.41 the model for males had an MSE of 0.35. Attractiveness scores in our sample averaged 3.35 [*SD* = 0.26] for males and 3.24 [*SD* = 0.28] for females on a scale from 0–4.

### Analyses

#### Convolutional neural networks

Deep learning approaches typically require vast amounts of information for effective training. Studies using sample sizes similar to ours often rely on pre-trained networks built on exposure to millions of example images, which researchers then fine-tune to the particular application of the study^29^. We also employ data augmentation, where we randomly resize and add noise to the existing images, which in essence corresponds to highly increasing the number of training examples at our disposal^30^. The pre-trained network used in the present study is VGG 16^30^. We use the training samples (which as noted above represent 70% of our full sample *N*) to fine-tune the network separately for males and for females. The existing learned parameters within VGG 16 (termed “weights” in deep learning research) are used and refined in the process of trying to predict a new outcome, in our case whether an image represents a left- or right-wing individual. The validation samples (15% of the full sample *N* for each gender) help prevent overfitting, where a model closely fits the training data but has little ability to predict outside of the training sample. Specifically, we select the set of weights developed within the training sample that show the highest accuracy in the validation sample. These weights are then used to predict ideology within the test sample, much like how regression weights in an ordinary least squares (OLS) regression can be used to calculate individual predictions for a given individual in a sample. Since we already know the ideology of these candidates, we can calculate how accurate the predictions are by comparing the predictions to the actual values.

The deep learning architecture we use is a convolutional neural network (CNN). A CNN uses multiple *local* features in an image instead of using all individual features in an image independently^31^. Simplifying somewhat, a CNN extracts small pieces of information from the total image and uses these local “snapshots” as features in the neural network instead of inputting all the information in the image as individual features at once.

Consequently, CNNs have two important characteristics^29^. First, the learned patterns are translation invariant, such that if a particular pattern is learned in an image, the neural network will be able to recognize it in another image even if this feature is not located in the same location in the second image. Second, CNNs learn highly abstract (and hierarchically ordered) features. Thus, we do not expect our CNNs to learn specific features (e.g., a large nose) as predictors of political ideology. Instead, they learn only abstract features that are a combination of a tremendous number of small features—the VGG 16 network we use consists of roughly 140 million parameters. This approach aligns with previous research on facial features in the social sciences, which finds that it is the combination of many features that provide the most useful information^11^.

#### Heat maps

CNNs have an important advantage over other deep learning architectures. They can produce visual indications—heat maps—of what they learn from^29^. Consequently, CNNs are less of a “black box” because they allow us to see which parts of an image a network focuses on when making predictions.

These heat maps have two distinct purposes in the present study. First, they allow us to ensure that the CNNs are attending to the intended elements of the image. Given the present study’s focus on faces, it would be problematic if heat maps showed the CNN was heavily influenced by parts of the image that don’t contain a face, such as the corners of the box that surrounds the face. Second, heat maps provide an informal tool to explore which features of a face are the most informative—for example, are all elements of the face providing important information, or is the CNN particularly attending to one feature or another? This information should facilitate further theory generation.

## Results

### Heat maps and non-facial information

**Figure 3:**
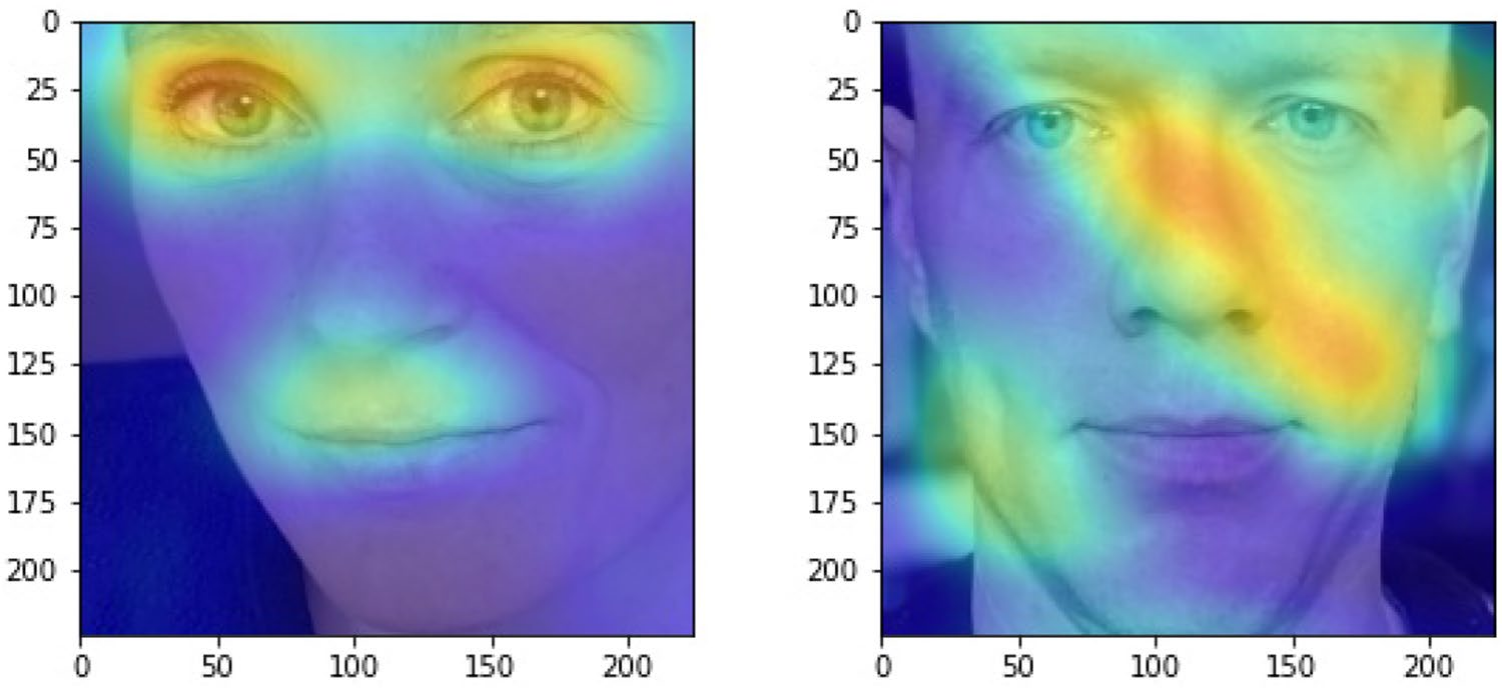
Illustrative heat maps for males and females. *Note*: These pictures represent where the CNN paid attention for females and for males—specifically, the average grad cam results for male and female left-wing candidates. Grad cam images for right-wing candidates can be found in Supplementary materials 1 Fig. 2.

Figure 3 shows the average heat maps for all male and female candidates in the test sample. These are then superimposed on two images to illustrate *which* areas in the face are most important in terms of making a prediction for left-wing and right-wing candidates.

**Figure 4:**
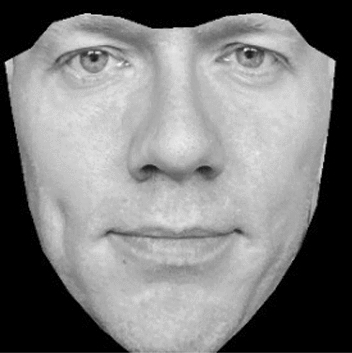
Illustration of male photo with background removed.

The preprocessing of images is performed as described above, and illustrated in Fig. 1—i.e., with original photos cropped to a small square with the face in the center, the face rotated, and the image gray scaled. Heat maps for females gave no cause for concern, as classification of females as liberal or conservative by the CNN is overwhelmingly done based on facial features. For males, however, elements beyond the face were substantially informing the classification. In particular, the presence, absence, or characteristics of shirt-collars appeared to be consequential. All results reported in the manuscript therefore use male images which were cropped a second time to exclude all non-facial elements from the photo (see Fig. 4).

### Predicting ideology using neural networks

Results on the full primary sample provided accuracies of 61% both for males and for females. These numbers are normed such that using a coin-flip to predict the ideology of the person in the image would be expected to succeed 50% of the time. Our models are therefore better at predicting ideology than a random guess. The accuracy results for males were affected by our exclusion of non-facial information. When our CNN was allowed to use versions of the male photographs (for training, validation, and test) that did not exclude non-facial elements—i.e., the version represented in Fig. 1—the accuracy was higher (65%).

To assess the replicability of our results we used the same CNN weights obtained after training and validation in the primary sample to predict ideology in our replication sample of Danish parliamentarians. For males in this sample we removed background information from the photo as described for the primary sample in order to include information not related to the face. In this sample we obtained accuracies of 61% for females and 57% for males, which is comparable to accuracies obtained in our primary sample. We now turn to exploring what has enabled the CNN this degree of success in identifying the ideology of participants based only on their face.

### Identifying salient characteristics

#### Facial averages

**Figure 5:**
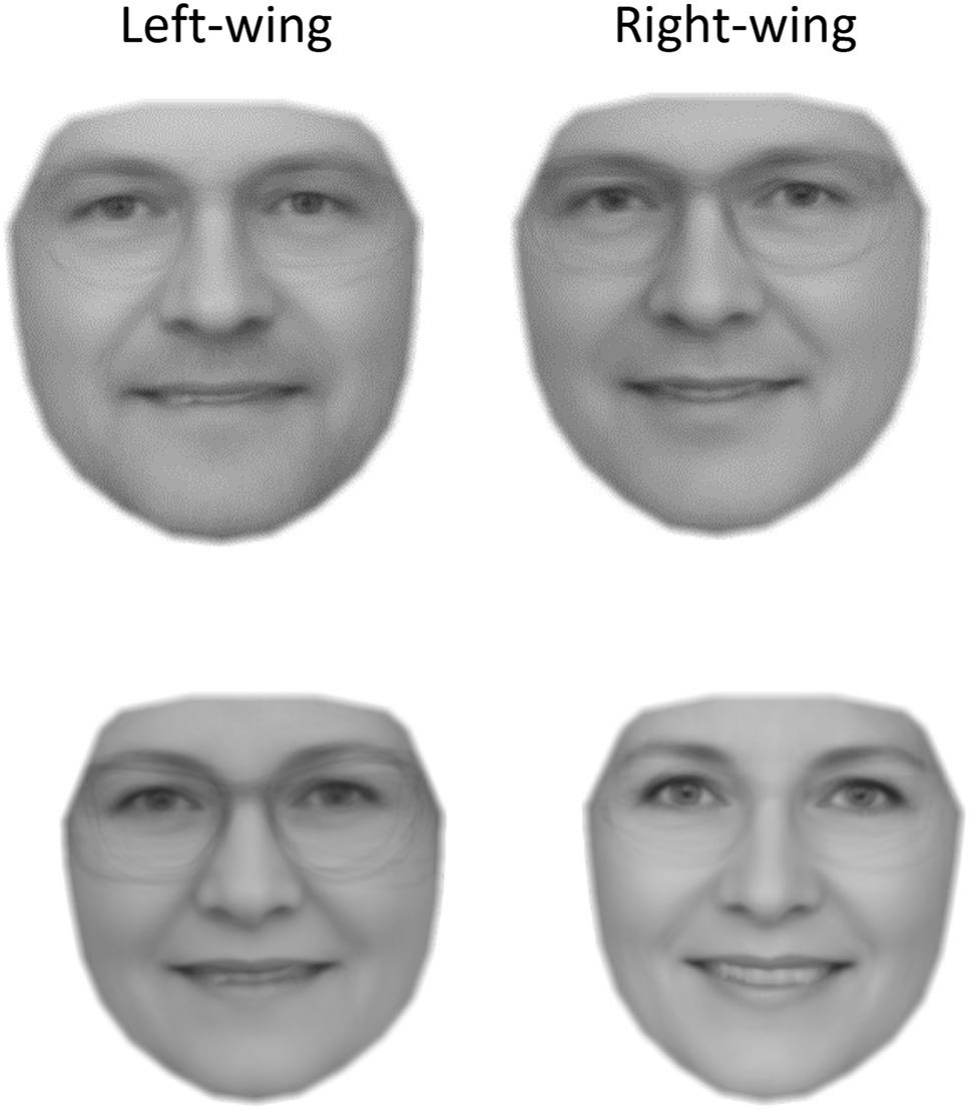
Facial averages for males and females Note. Facial averages for the candidates with the 20 most extreme scores on predicted ideology for each gender. E.g., the facial average of the 20 male candidates with the most extreme predicted left-wing beliefs are top left.

We created facial composites^5^ for individuals identified by our model to be most likely to be left- or right wing. We do this to investigate if facial features recognizable by the human eye are responsible for the predictive accuracy of our model. Figure 5 represents the four resulting facial averages, with the top left face, representing the average of the 20 male candidates our model identified as most likely to be left-wing. Most clearly we see that both male and female right-wing composites appeared happier than their left-wing counterparts. The male composites were not obviously differentiated based on masculinity. The right-wing faces, particularly for females, might be perceived as more attractive.

#### Morphology

Consistent with the impression from facial composites, masculinity scores were unconnected to model-predicted conservatism [*r* = 0.02, 95% CI [− 0.10; 0.04]]. All reported correlations are Spearman correlations to account for the fact that many of our measures are not normally distributed but quite skewed. Attractiveness was not significantly correlated with model-predicted conservatism in males [*r* = 0.03, 95% CI [− 0.033; 0.088]]. By contrast—and also matching the impression conveyed by facial composites–females attractiveness scores positively correlated with model-predicted conservative ideology [*r* = 0.11, 95% CI [0.042; 0.178]].

#### Expressions

Consistent with informal impressions from the facial averages, happy facial expressions were associated with model-predicted ideology. The predicted probability of a male face being right-wing correlated 0.17 (95% CI [0.11, 0.23]) with happiness and − 0.17 (95% CI [− 0.23; − 0.11]) with neutrality. The corresponding results for females were 0.15 (95% CI [0.08; 0.22]) and − 0.15 (95% CI [− 0.22; 0.08]).

That the results for happiness and neutrality are nearly exact reversals reflects the fact that other facial expressions were very infrequent in this sample. As noted above, the procedure used to score facial expressions appears to be less accurate beyond happiness and neutrality, and so interpreting results for other facial expressions require caution. However, we did observe that among women contempt was non-trivially correlated with a predicted probability of left-wing ideology (*r* = 0.12, 95% CI [0.05, 0.19]). No other relationship between facial expressions and ideology were statistically significant for either gender.

## Discussion

Our results confirmed the threat to privacy posed by deep learning approaches. Using a pre-developed and readily available network that was trained and validated exclusively on publicly available data, we were able to predict the ideology of the pictured person roughly 60% of the time in two samples. We also found that existing practices, in which non-facial information is typically included in the small boxes surrounding a face, could produce inflated accuracies: Heat mapping results showed our CNN was originally using information other than the face to classify males. Had we not deleted this information our accuracy would have been higher.

We also provide the first demonstration that model-predicted ideology connects to independently classifiable features of the face. For females (though not males), high attractiveness scores were found among those the model identified as likely to be conservative. These results are credible given that previous research using human raters has also highlighted a link between attractiveness and conservatism^10^. Recall that we use photographs provided by the candidates themselves while campaigning for (low-level) political office. Because attractiveness generally helps electoral success, all candidates are incentivized to provide an attractive photograph^10^. Further, the amateur nature of these candidates is a strength because the photos represent candidates for office where selective pressures are rather low. Consequently, we are less (which is not to say zero) concerned that the relationship results from a particularly intensive selection of attractive candidates among those on the right.

Attractiveness was not the only correlate of model-predicted ideology. We also found that expressing happiness is associated with conservatism for both genders. Previous work has found smiling in photographs to be a valid indicator of extraversion^32^, and while extraversion is not broadly associated with ideology^33^, some studies have found that right-wing politicians are more extraverted^34^; though see^35^. Self-presentation should also be considered. Politicians on the left and right may have different incentives for smiling—for example, smiling faces have been found to look more attractive^36^, which is comparatively important for conservative politicians. Future work is needed to explore the extent to which happy faces are indicative of conservatism outside of samples of politicians.

While the use of a politician sample represents a potential constraint on generality, the use of candidates for local office in Denmark, attenuates this constraint. First, because selective pressures are relatively small on this candidates and secondly because prior work on attractiveness and politics reports similar findings across different highly developed political contexts^10^.

Future work might explore why our heat maps showed the eyes and mouth regions to be important for the classification of female ideology. While the relevance of these regions to identify things such as whether a face is happy may seem straightforward, happiness correlated with model predicted ideology similarly for males and females, so why a given facial region would be more diagnostic for one gender than another is not immediately obvious.

## Supplementary Information

Supplementary Information.
