# Supplementary material for: Using deep learning to predict ideology from facial photographs: expressions, beauty, and extra-facial information
Source: Sci Rep. 2023 Mar 31;13:5257. doi: 10.1038/s41598-023-31796-1 (PMC10066183; doi:10.1038/s41598-023-31796-1)
Supplement: Supplementary file 1 — Supplementary Information. [file 41598_2023_31796_MOESM1_ESM.docx]

**Supporting information 1: Additional results**

The parties were split by left and right based on their self-identified current ideological stance in Danish politics. Leftist parties are: the Unity List, The Socialist People's Party, The Danish Social Liberal Party, The Alternative, The Social Democrats, and the Rightist parties are: The Conservative People's Party, The Danish People's Party, Denmark's Liberal Party, Liberal Alliance and The New Right.

*Table 1: Test, training and validation data splits*

|  | **Training**  **(blue/red)** | **Validation**  **(blue/red)** | **Test**  **(blue/red)** |
| --- | --- | --- | --- |
| **Male** | 566/471 = 1108 | 117/82 = 199 | 99/70 = 169 |
| **Female** | 352/458 = 810 | 87/94 = 181 | 67/97 = 164 |

*Figure 1: Two examples of grad-cam technique picking up on shirts*

**
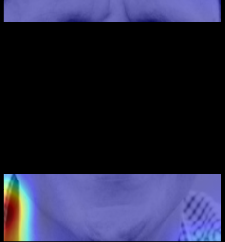

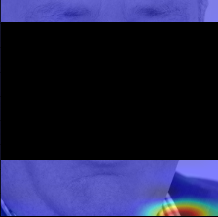
**

Figure 2: *Illustrative Heat Maps for Males and Females*

**
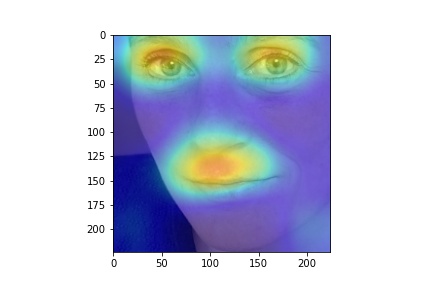

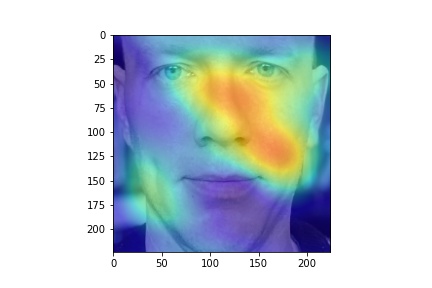
**

*Note*. These pictures represent where the CNN paid attention for females and for males for right-wing candidates.

**Supporting information 2: Formal description of CNN network**

This formal description of CNNs is based on Goodfellow et al. (2016). The classical deep learning network is the multilayer perceptron (MLP) network. This type of network is called a "deep" learning network because it consists of one or more so-called "hidden layers."

More formally we can define the mapping $y=f(x;\theta)$ where y are items to be classified, *𝑥* is our input, and $\theta$ represents our parameters. We want to train the network to get as close as possible to the "true" function *𝑓* ^∗^ governing the data generating process.

The idea is to stack multiple layers on top of each other, e.g., $f(x) = f^{3}\left( f^{2} \right)(f^{1}\left( x \right))$). To fix ideas let us define a network with the parameters *𝑤* and *𝑏*. For each layer we add an activation function, e.g., a rectified linear activation function (ReLU), which can be defined as an activation function $g(z) = max\{0,z\}$. If no activation function is employed the network is not able to learn nonlinear functions of the data which is one of the huge benefits of neural networks, namely its ability to learn highly nonlinear and complex relationships.

By adding this function each layer now has the form: $relu(x^{T}w+b)$. This feeds the output from the previous layer to the next in the form of a series of dot products. The final output layer transforms the output from the last layer into a probability of predicting the outcome class of interest using a logistic function. The final output of the network needs to correspond to the classification task at hand, which in our case is a simple binary classification task. Consequently we simply use a logistic function as our output layer in this analysis.

Training the network consists of three parts. First, all weights are randomly initialized to some small value. Second, the network calculates all values, i.e., outputs from the network based on the (initialized) weights and the inputs *𝑥*. To update the weights the technique of backpropagation is used (McClelland, Rumelhart, Group, et al. 1986). Backpropagation starts out by calculating the loss for each iteration in our network. Since we have a binary classification problem the loss function is simply a log-loss cost function which can be written as:

APSRSubmissionTemplateAPSRSubmissionTemplateAPSRSubmissionTemplateAPSRSubmissionTemplateAPSRSubmissionTemplateAPSRSubmissionTemplateAPSRSubmissionTemplateAPSRSubmissionTemplate

$$J(\theta) = -E_{x,y\sim\hat{p}_{data}}\log p_{model}(x|y)$$

Based on the loss, the weights are then updated sequentially from the last layer to the first.

The novel thing about convolutional neural networks (CNN) is the way they define the layers. First, a CNN uses so-called convolutional layers, which are a series of small linear layers which slide over the input image and produce a single output. Instead of using the entire output of this convolutional layer only a slice of this is used. If we call this function S and let it operate on an image of size *m* x *n*, with a kernel of size *i* x *j* this can be written as

$$S\left( i,j \right)=\sum_{m} \sum_{n} I(m,n)K(i-m,j-n)$$

These convolutional layers are the main drivers of convolutional neural networks. Typically in a convolutional neural network, the outputs of a convolutional layer is input to additional layers, as well as additional convolutional layers, so the specific layout of a convolutional neural network is quite complex and can be defined in a multitude of ways.
